# Dyke intrusion and stress-induced collapse of volcano flanks: The example of the 2018 event at Mt. Etna (Sicily, Italy)

**DOI:** 10.1038/s41598-020-63371-3

**Published:** 2020-04-14

**Authors:** E. Giampiccolo, O. Cocina, P. De Gori, C. Chiarabba

**Affiliations:** 1 INGV, Sezione di Catania - Osservatorio Etneo. Piazza Roma, 2 – 95125 Catania, Italy; 2 INGV, Osservatorio Nazionale Terremoti. Via di Vigna Murata, 605 - 00143 Roma, Italy

**Keywords:** Natural hazards, Solid Earth sciences

## Abstract

Magmatic intrusions, eruptions and flank collapses are frequent processes of volcano dynamics, inter-connected at different space and time scales. The December 2018 recrudescent episode at Mt. Etna is an exemplary case where a sudden intrusive event culminated with a short eruption, intense seismicity and a shallow large strike-slip earthquake at the edge of the eastern sliding flank. Here, we show that high resolution velocity models and transient changes of V_P_ and V_P_/V_S_ resolve the magma intrusion through a dyke and local stress increase at the base of the unstable flank, inducing the collapse. Episodic brittle faulting occurs at the edge of the sliding sector, locally contributed by high fluid pressure. The feedback between magma ascent, stress changes and flank collapse is driving the volcano dynamics, with processes ranging from long term to transient episodes.

## Introduction

Earthquake and volcanoes are expression of dramatic changes in the dynamics of the earth interior, connected at a scale that ranges from plate tectonics to local^1,2^. The relation between magmatic intrusions, flank collapses and earthquakes is still an open issue, although evidences at paradigmatic giant strato-volcanoes are progressively elucidating some main aspects^3–6^.

The continuous magmatic activity and deformation make Mt. Etna (Sicily, Italy) one of the most intriguing sites for testing the ability to model and predict the behaviour of complex dynamic systems. Long term processes of deep magma recharge and storage within the upper crust^7,8^, sudden dyke intrusions and eruptions^9–11^, flank collapses with abnormal velocities [e.g.^12,13^ and references therein] represent the standard evolution of Mt. Etna.

**Figure 1:**
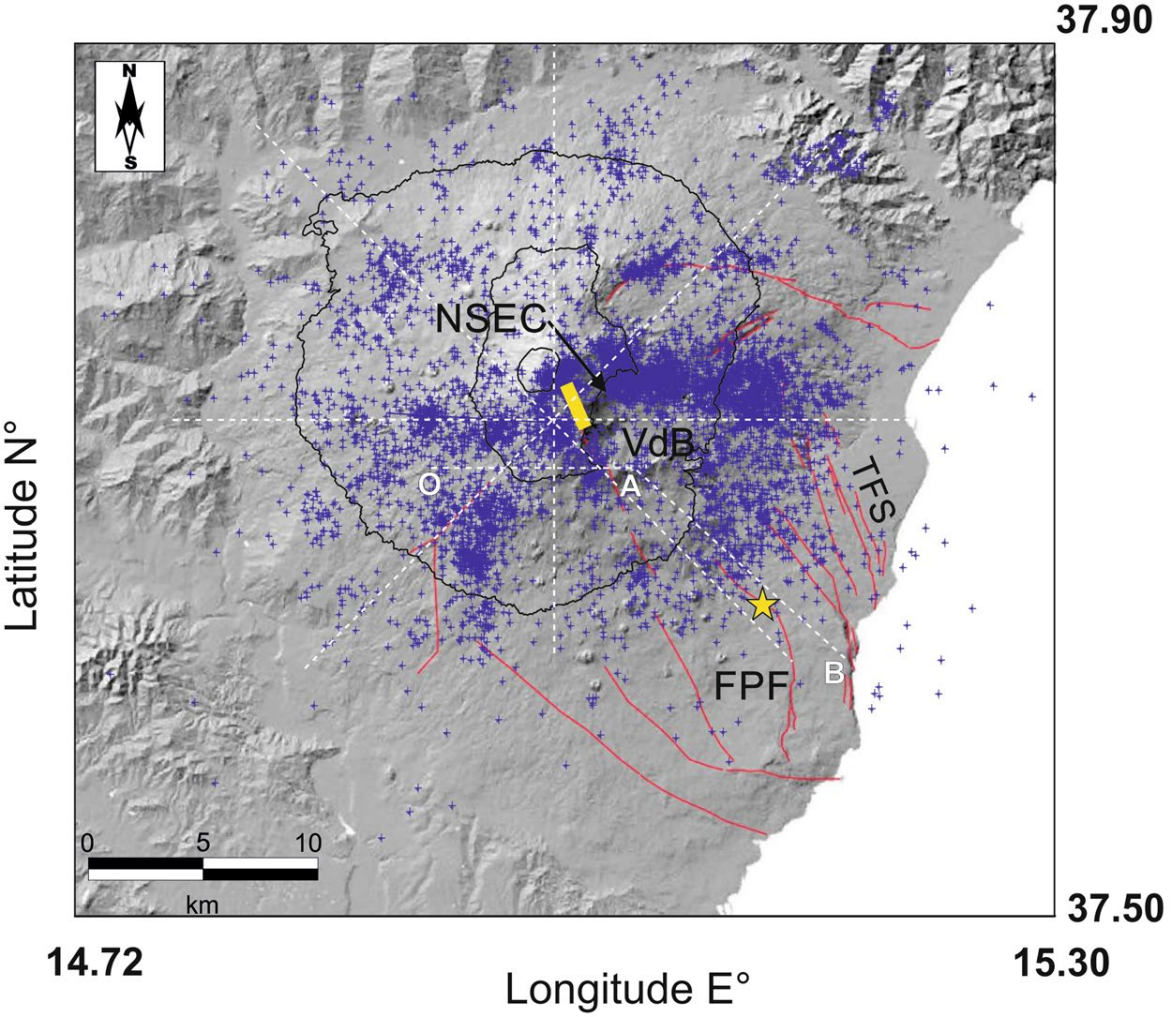
Map of Mt. Etna volcano (Sicily, Italy) seismicity (blue crosses) and main tectonic elements [red lines^14,15^]. FPF: Fiandaca-Pennisi Fault; TFS: Timpe Faults System; NSEC: New South-East Crater; VdB: Valle del Bove. Yellow thick line indicates the eruptive fissure opened on December 24 [modified after 30]. The yellow star indicates the epicentre of the December 26 M_W_ = 4.9 earthquake. The white dashed lines are the traces of the profiles shown in Fig. 3. The shaded image of the DEM is from^44^, reprinted by permission of the publisher (Taylor & Francis Ltd., http://www.tandfonline.com).

The 2018 episode of recrudescence is an exemplary case of strato-volcano dynamics. On December 24, deformation and seismicity accelerate accompanying an episode of magma intrusion and eruption from the summit area, culminating, a few days later, with a magnitude M_W_ = 4.9 earthquake on the Fiandaca - Pennisi Fault (hereafter FPF; Fig. 1). This SE-trending fault runs along the middle-lower flank of the volcano as observed in Fig. 1, as part of the broad set of faults composing the Timpe Faults System [TFS^14,15^]. While most of the seismicity clustered around the intruding magma (Fig. 1), this large earthquake, the strongest since decades, occurred isolated and not followed by aftershocks. During its recent history, lateral eruptions showed similar connection between magma intrusion within the shallow plumbing system and the, prevalently aseismic, collapse of the eastern flank [e.g.^16–21^]. Anyway, reciprocity in cause/effects is still widely debated. Flank instability and sector collapses are rather common during the evolution of a volcanic edifice, and may happen at any volcano [^22^ and references therein]. Prominent examples are those in the Hawaiian, Canarian, Cape Verdean and Reunion archipelagos [^5^ and references therein].

In this study we investigate the relation between magma intrusion and flank instability, as highlighted by the distribution of seismicity within the volcano plumbing system and transient changes in elastic parameters. We compute 3D and 4D tomographic models (i.e. in time and space) by using a comprehensive data set of P- and S-wave arrival times from past-decade seismicity. The present V_P_ and V_P_/V_S_ images have a high resolution from the surface down to 18 km depth, enhancing the imaging of previous models^19,23–25^. The persistent seismicity permits to resolve time-lapse velocity images relative of the 2018 intrusive period (December 2018–January 2019). Data and methods used in the work are reported at the end of the paper.

## Results

### velocity images

**Figure 2:**
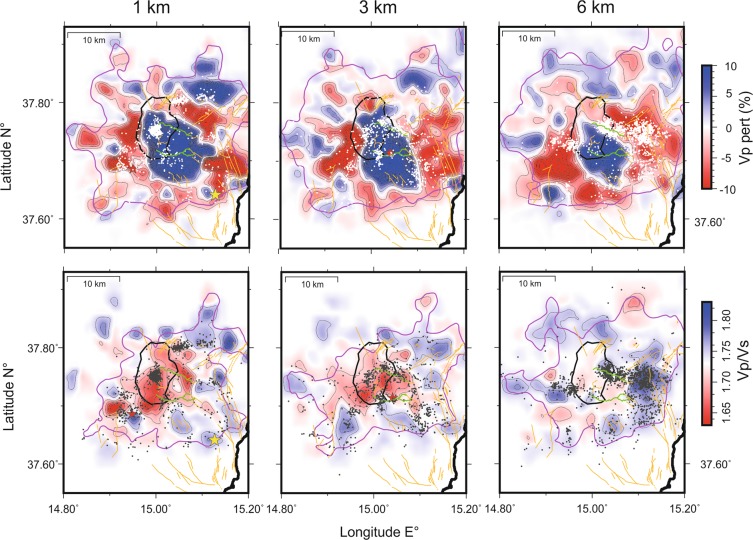
Layers (depths in km b.s.l.) of the present V_P_ (% of perturbation) and V_P_/V_S_ models describing the shallow portion of the volcanic system (depth refers to sea level). The limit of the well resolved regions (SF ≤ 3.0) is shown by the purple line. Relocated earthquakes are plotted in white (V_P_ layers) and grey (V_P_/V_S_ layers) dots (+/− 1 km from each layer); Red stars are M_L_ ≥ 4.0 earthquakes and yellow star is the December 26 M_W_ = 4.9 earthquake. Structural elements are reported in orange [from^15^]. The green lines are the limits of the Valle del Bove (VdB).

**Figure 3:**
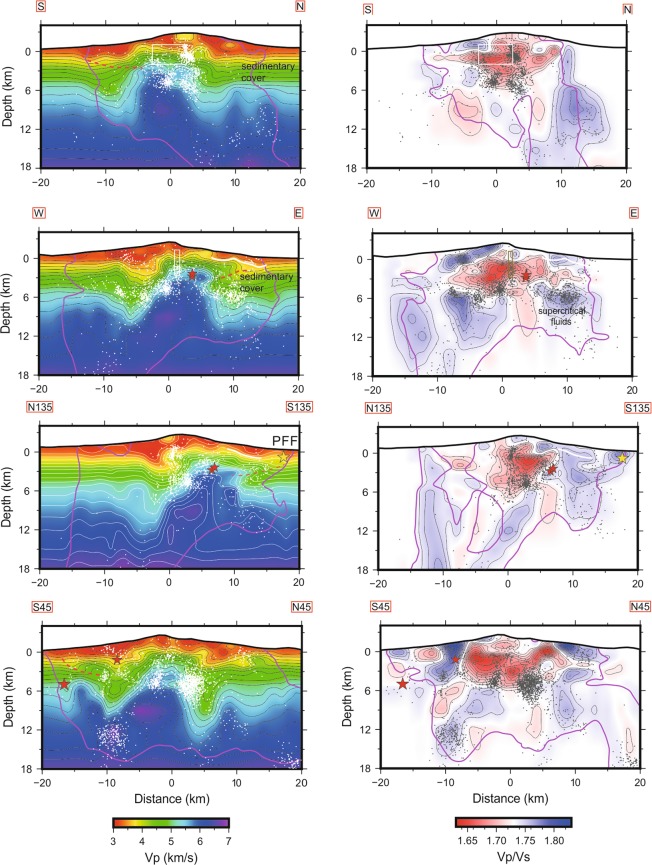
Vertical sections of V_P_ and V_P_/V_S_ along the traces reported in Fig. 1 and relocated seismicity across the volcano. Purple lines delimit the well resolved regions (SF ≤ 3.0). Red stars are M_L_ ≥ 4.0 earthquakes and yellow star is the December 26 M_W_ = 4.9 earthquake. The box indicates the extent of the intrusive dyke [from^30^]. Dashed red lines mark the compressional structure within the sedimentary cover, as retrieved from V_P_ anomalies. The white line indicates the decollement layer, at the top of the sedimentary units.

The 3D velocity images obtained present strong heterogeneities in V_P_ and V_P_/Vs, shown as perturbations in horizontal layers (Fig. 2) and absolute velocity in vertical sections (Fig. 3). The main morphological features in the 3D images are:

- A huge high V_P_ body (HVB hereafter) located in the central-southern part of the volcano and extending toward the eastern flank, resolved from the surface down to 18 km depth. This body, generally observed in all previous studies [e.g.^19^ and references therein^25^], is superiorly resolved in terms of refined pattern and geometry. Seismicity mostly occurs around HVB, except clusters beneath the summit area that occurred at its western border. The upper part of the HVB has persistent low V_P_/V_S_ anomalies. Following^23^, the HVB can be interpreted as a massive accumulation of intrusions pervading the sedimentary basement, that repeatedly were emplaced during the recent and past activity of the volcano [see also^26^].
- Low V_P_ anomalies around the central intrusive mesh (down to 9–12 km depth), representing the sedimentary substratum deformed with southward verging system of thrust nappes of the Apenninic–Maghrebian Chain^27^. The association with high V_P_/V_S_ anomalies suggests the existence of wide volumes over-pressured by fluids within the sedimentary units around the volcano. Usually, low V_P_ and high V_P_/V_S_ anomalies are associated with fluid-filled pressurized rock volumes, since the attitude of the pore pressure to force cracks to remain open^28^.
  Seismicity is abundant in many of these volumes suggesting intense phenomena of micro-cracking assisted by the high pore pressure. We hypothesise that these volumes contain significant portion of supercritical fluids generated by interaction between regional aquifers contained within the sedimentary cover and the central still hot portion of the intrusive body;
- Lack of distinct low V_P_ and high V_P_/V_S_ anomalies indicative for the presence of significant magma volumes stored in the upper crust. In general, melts and fluids are associated to abrupt drops in shear wave velocity, and therefore to high V_P_/V_S_ anomalies^29^.
  Anyway, the absence of seismicity within the deep portion of the intrusive mesh could indicate very high temperature and, possibly, the stocks of small magma batches at depths below 6 km;
- Shallow low V_P_/V_S_ anomalies suggesting the presence of broad gas-dominated volumes within the upper portion of the body, since the decrease of density by free gas in pores induces an increase of shear wave velocity and a decrease of V_P_;
- A gentle warping of V_P_ anomalies at the edge of the volcano, that we interpret as a compressional structure within the sedimentary cover [see^27^], developed during the belt formation (red dashed lines in sections of Fig. 3). This originally north-dipping set of thrust units result uplifted and tilted by the successive emplacement of the HVB. We hypothesize that this set of faults branching from a deeper main thrust might form preferential weakness for the collapse of the volcano flanks.

**Figure 4:**
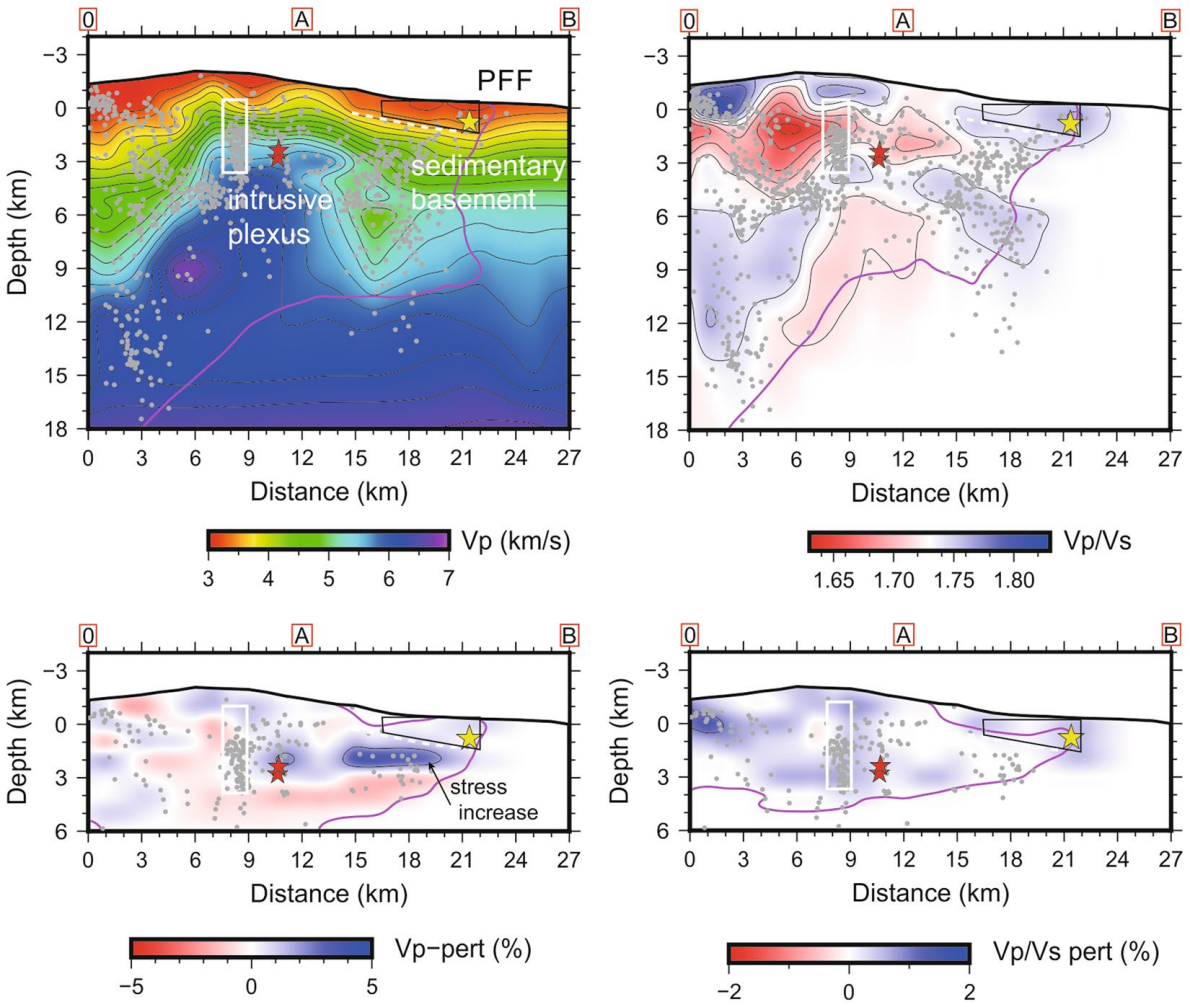
Vertical section of V_P_ and V_P_/V_S_ (top) across the 2018 intrusive dyke (segment O-A in Fig. 1) and along the lateral collapse fault (segment A-B in Fig. 1). The white box indicates the extent of the intrusive dyke, while dashed white line indicates the decollement layer. Red stars are M_L_ ≥ 4.0 earthquakes and yellow star is the M_W_ = 4.9 event. The lower panel shows V_P_ and V_P_/V_S_ changes during the intrusive period. Note the increase of V_P_ (increase of stiffness) around the intruded dyke that propagates at the base of the decollement. High V_P_/V_S_ on top of the decollement and along the FPF (black polygon) is observed, as well as an increase during the intrusion.

In addition to these robust elements of the volcano structure, peculiar transient changes in V_P_ and V_P_/V_S_ are revealed during the 2018 episode (Fig. 4) by the time lapse imaging (see the Data and Method section for technical details). Although the difference in velocities between the eruptive and the entire period may partially depend on a different sampling of the volcano structure by seismic rays, some main features appear to be located in volumes that are similarly resolved in time. In this case we are confident that the transient change is reliable, especially when V_P_ and V_P_/V_S_ changes are of different sign. The main features that we comment are:

- A relative decrease of V_P_ and increase of V_P_/V_S_ within the HVB and in the volume of the intruded dyke, that is consistent with upraise of melts at shallow depth;
- A relative increase of V_P_ around the intruded dyke and at the base of the decollement layer;
- A relative increase of V_P_/V_S_ along the south-eastern flank and in correspondence of the FPF, suggesting increase of pore pressure along this shallow fault system.

The most interesting transient anomaly is the V_P_ increase at the base of the decollement layer. This anomaly is well pronounced and connected with a general increase of velocity around the intruded dyke. Although different factors may influence this increase, we are attracted to explain it with an increase of stress produced by the intruded dyke.

### Magma intrusion and flank collapse

Information on the deep structure of Mt. Etna and transient changes of velocities highlight how the volcano reacts to intense episode of deformation associated with dyke intrusion. Seismic swarm accompanied the intrusion of a N-NW oriented shallow dyke^30^ which splays from the summit area down to few kilometres below the sea level, on the top of the high velocity intrusive complex. The shallow portion of the volcano structure, comprising the central high V_P_ plexus, is characterized by broad low V_P_/V_S_ anomalies, which we interpret as gas-filled volumes. The absence of clear low V_P_ and high V_P_/V_S_ anomalies argues against the presence of significant shallow magmatic volumes. A volume with such anomalies has been never identified beneath the volcano^19,23–25^, while broad high attenuation anomalies have been interpreted as high temperature volumes, possibly constituting the principle paths of magma upraise around the HVB^31^. The tendency of seismicity to be absent in the deeper part of the HVB (Fig. 3) might point to the existence of high temperatures and small magma batches at a depth that is consistent with that of depleted source argued by geodetic modelling [e.g.^32^].

During the dyke intrusion, deformation spread over the eastern flank^30^. We observe distinctive time-change of velocity associated with the magmatic intrusion. While V_P_ decreases and V_P_/V_S_ increases within the intruding dyke, consistently with a local magma upraise, the P-wave velocity is higher east of the dyke (Fig. 4). In our hypothesis the increase of V_P_ is related to an increase of stress caused by the dyke intrusion. The stress concentrated at the base of the weakness zone, along which the flank is collapsing, which position and depth is defined by modelling of geodetic data^33^. Edge faults splaying from the decollement and bisecting the eastern sector of the volcano, like the FPF, were mobilized during the flank sliding.

The large volcano-flank earthquakes, among which the M_W_ = 4.9, tend to occur within low V_P_ and high V_P_/V_S_ volumes (Fig. 2), reinforcing the idea that over-pressured fluids play a major role in triggering seismicity, as also suggested by the transient increase of V_P_ and V_P_/V_S_ observed close to the earthquake hypocenter (Fig. 4).

On a broad scale, seismicity abounds within low V_P_ and high V_P_/V_S_ zones located around the volcano, testifying for intense cracking of fluid over-pressured rock volumes^28^. This supports the existence of a wide crustal volumes filled by super-critical fluids around Mt. Etna volcano. We are attracted to hypothesize that the long term breath of this giant deep hydrothermal system concur in the dynamics of the volcano.

## Conclusions

Tomography and time-lapse imaging of active volcanoes are fantastic tools to explore volcano dynamics. We observe changes in velocities during an eruptive period at Mt. Etna that highlight stress changes caused by dyke intrusion. Magma intruded within the sedimentary cover on top of the broad intrusive mesh, generating a transient velocity increase consistent with stress propagation on the unstable flank. The collapse of the volcano flank is favoured by pre-existing structure within the sedimentary cover, revealed by tomography, that acts as the main decollement during the sliding. This structure acts also as an impermeable barrier for fluids favouring local over-pressure at the base of the high angle faults present along the volcano flanks.

To clarify the role of the wide hydrothermal system in triggering transient episodes of deformation and magmatic supplies remains challenging and urgent. This can be of evaluable importance for characterizing the volcano dynamics and forecasting changes in activity.

### Data and methods

We used earthquake data recorded in the period 2005–2019 at 30 seismic stations equipped with broadband (0.01–40 s) three-component seismometers, yielding a homogeneous sampling of a wide volume, including the central craters, the flanks and the deep portion of the plumbing system (Fig. SOM1). P- and S-wave arrival times, manually read on digital waveforms, and earthquake location parameters were selected from the INGV-OE catalogue^34–36^. Hypocentral locations were obtained by using the Hypoellipse code^37^ and the 1D V_P_ velocity model optimised for the real time monitoring^38,39^, with a starting V_P_/V_S_ = 1.73, derived from Wadati regression of the P and S arrival times. Then, we selected the best located events having at least 6P and 2S arrival times, RMS ≤ 1 s, hypocentral errors ≤1.0 km and azimuthal gap ≤180 degrees. To enhance the recovering of structures to the east of the summit area, we used a few events with azimuthal gap up to 200 degrees but with hypocentral solution well constrained by nearest station located within 5–10 km. This selection provided a total of 7306 earthquakes with 90481P travel times and 28532 S-P differential times. To perform the tomographic inversion, we used the SimulPS14 code^40^, which is an extension to the widely used Simul-code family, originating from^41^. It is based on linearized iterative approach that solves for V_P_ and V_P_/V_S_ parameters on a 3D grid of nodes, where the velocities are continuously defined within the volume by a trilinear interpolation function. Hypocentral locations are simultaneously determined and updated at each iteration step, while V_P_ and V_P_/V_S_ parameters are computed, after parameter separation, by inverting P and S-P times with damped least-squares algorithm. The tomographic model has been parameterized with nine, 1 km spaced, layers from −2 km above the sea level down to 6 km depth, to enhance the imaging of the shallow system, and five deep layers, 3 km spaced, bottoming at 30 km depth. Nodes are spaced every 2 km, horizontally. This parameterisation, selected after running several inversion tests with different node spacing, represents the best compromise between data misfit reduction, spatial model definition, and formal resolution of parameters expressed by the resolution matrix. We set damping values of 100 and 200 for the V_P_ and V_P_/V_S_ inversions, respectively, based on the analysis of the damping trade-off curves. After ten iterations, we obtained a final RMS value 0.128 s with a variance improvement equal to 67% and 21% for P and S-P data, respectively.

The reliability of the tomographic model has been estimated by the analysis of the resolution and covariance matrix. The resolution of each parameter was quantified by the spread function (SF) that measures the compactness of each averaging vectors of the resolution matrix. Small SF indicates well resolved node with averaging vector picked around its diagonal value and with negligible contribute of off-diagonal elements. To establish the threshold of SF below which the model resolution is adequate, we analysed the SF as function of the Derivative Weigh Sum (DWS), for each node. The plot usually displays a sort of L-shaped trend, with DWS decreasing as SF gradually increases, because the resolution is strongly dependent on the quality of sampling. The threshold for a reliable resolution corresponds to the SF value where the kink of the L-shaped curve is located (SF ≤ 3.0 in our case). In addition, the analysis of the covariance matrix demonstrates that well resolved nodes have formal errors less than 10 for velocity perturbations.

The vast dataset permits to compute tomographic time-lapse snapshots, to capture eventual transient anomalies distinctive of unrest episodes. Spatial and temporal uniqueness of seismicity in the intruding dyke guided us to use the approach proposed by^42^ rather than those based on even events^43^. The dataset is subdivided into two periods before and after the onset of activity in December 2018. In the first long-period, a total of 81547 and 25668P and S observations from 6679 earthquakes were inverted obtaining, after ten iterations, a variance improvement of 68% and 21% for P and S data, respectively. The overall model is similar to that obtained for the entire period inversion (Fig. S2). Using this 3D heterogeneous model as the starting model, we inverted the subset of 511 earthquakes recorded between December 2018 and January 2019, obtaining a variance improvement of about 32% and a final RMS of 0.14 s. This approach is thought to help time-lapse imaging, since the initial high definition of the structure compensates the minor sampling of the smaller dataset, while only significant changes are permitted. Details of data and models obtained by the entire and the subset inversions are shown in figures in the Supplementary Material.

## Supplementary information

Supplementary information.
