# Supplementary material for: Dyke intrusion and stress-induced collapse of volcano flanks: The example of the 2018 event at Mt. Etna (Sicily, Italy)
Source: Sci Rep. 2020 Apr 14;10:6373. doi: 10.1038/s41598-020-63371-3 (PMC7156433; doi:10.1038/s41598-020-63371-3)
Supplement: Supplementary file 1 — Supplementary information. [file 41598_2020_63371_MOESM1_ESM.pdf]

Online material Paper: **Dyke intrusion and stress-induced collapse of volcano flanks: The example of the 2018 event at Mt. Etna (Sicily, Italy)**

E. Giampiccolo[1], O. Cocina[1], P. De Gori[2] and C. Chiarabba[2]

1 INGV, Sezione di Catania - Osservatorio Etneo. Piazza Roma, 2 – 95125 Catania, Italy
2 INGV, Osservatorio Nazionale Terremoti - Via di Vigna Murata, 605 - 00143 Roma, Italy

This online material contains information about data and methods.

It consists of four main figures that illustrate details useful to address the reliability of computed transient variations of velocities among distinct periods. In particular we show:

Figure SOM1: The setup of the three different inversions computed in the study (All-period, Pre-Eruptive, Eruptive periods)

Figure SOM2-3-4: Velocity models computed for the three different inversions in representative layers and in the vertical sections reported also in the main text.

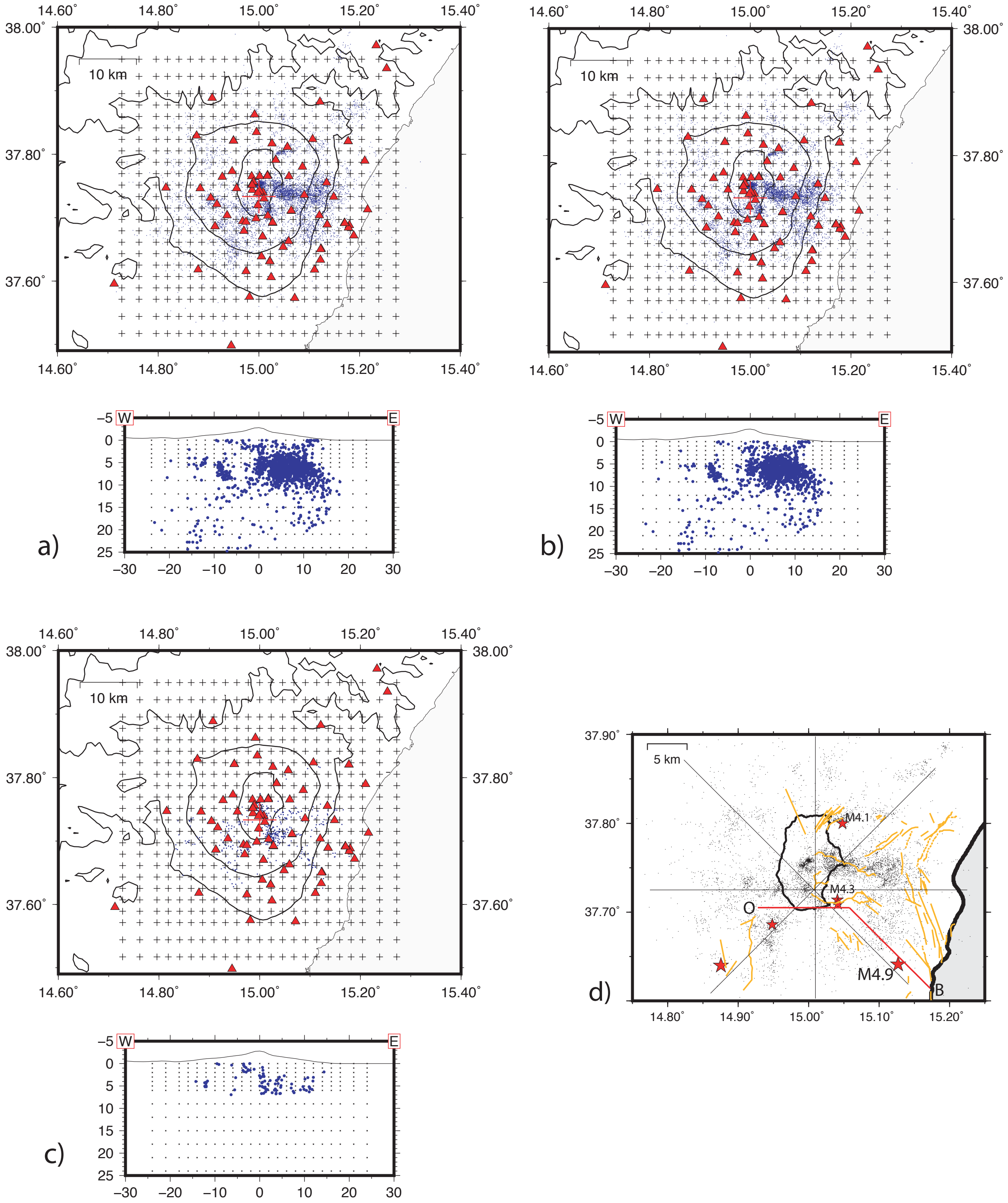

Figure SOM1: Set up in map and E-W cross section showing earthquakes stations and grid nodes used for the inversion of the All- (a), pre-eruptive (b) and 2018 eruptive (c) periods, respectively. Panel d shows the entire seismicity with M>4 earthquakes (stars) occurred since 2005 and the traces of the vertical sections.

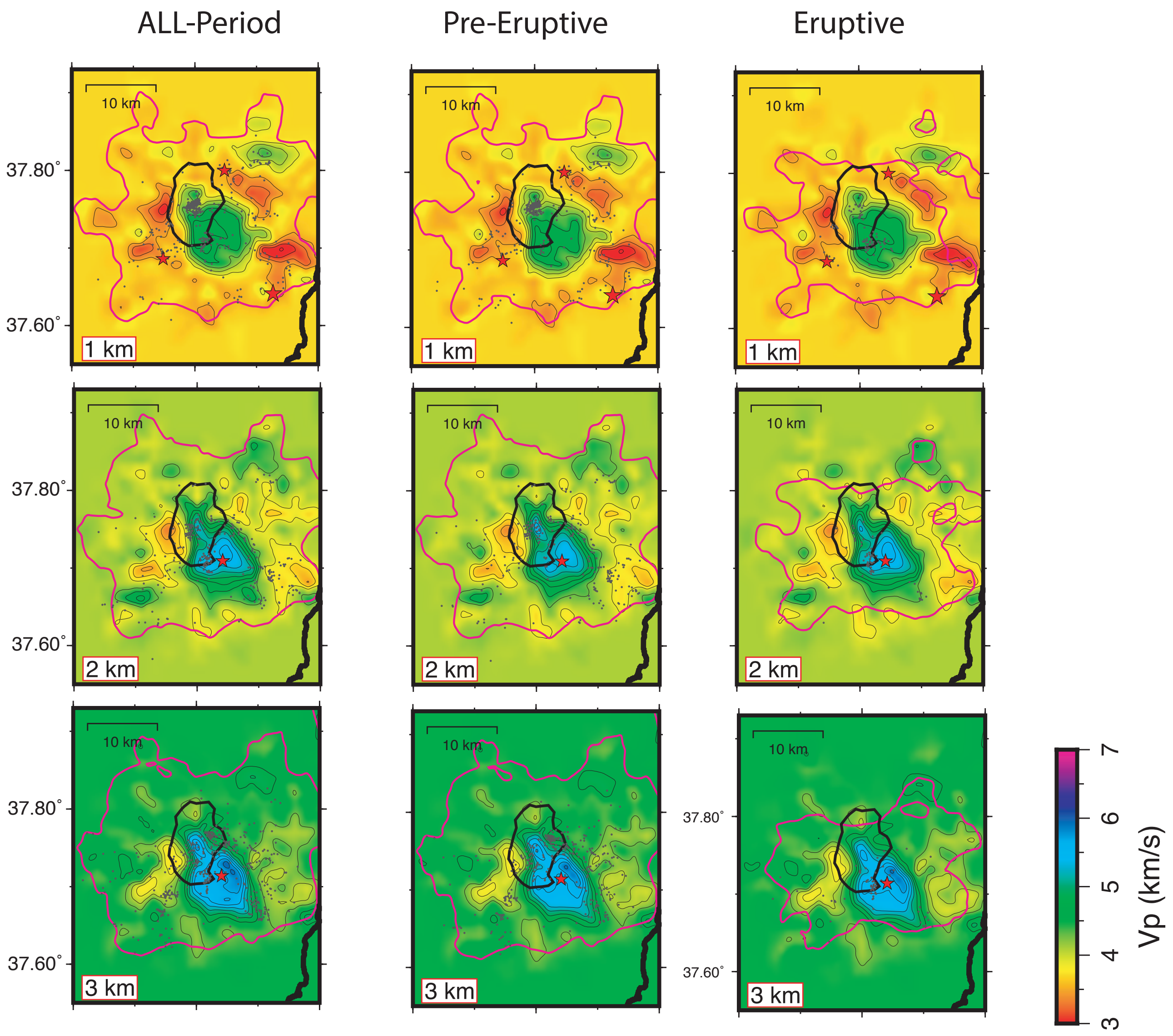

Figure SOM2: Vp models obtained for the three periods in layers at 1, 2 and 3 km depth. Earthquakes occurred at +/- 0.5 km from each layer are shown (stars are M>4). The topography isoline of 3000 mt is shown as a bold black line. Purple lines is the spf=2.0 indicating the well resolved regions in each inversion.

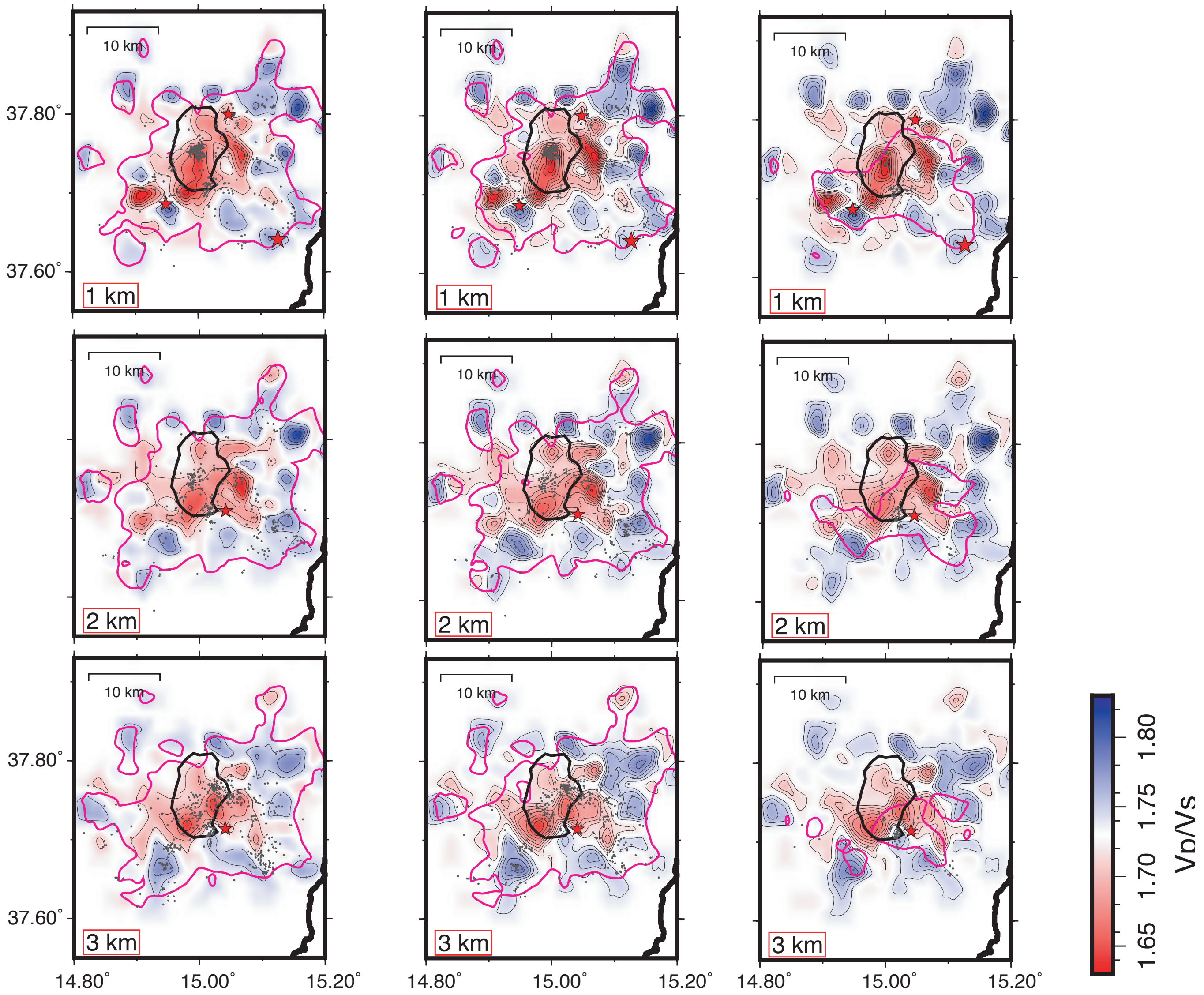

Figure SOM3: Same as Figure SOM2 but for Vp/Vs models.

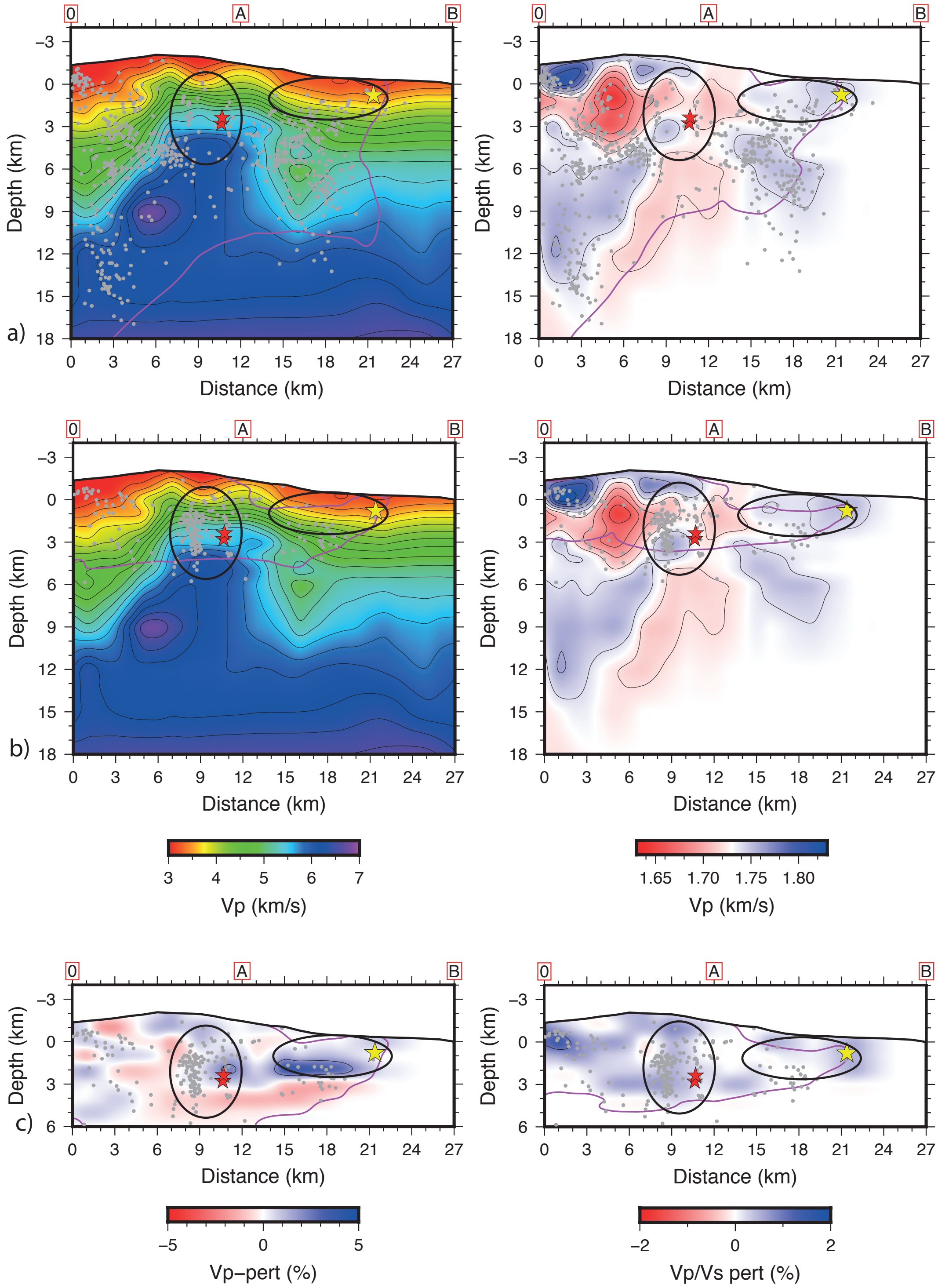

Figure SOM4: Vertical sections of Vp and Vp/Vs models along the profile O-A-B (see trace in figure SOM1) for the pre-eruptive (a) and the 2018 eruptive (b) periods and the velocity differences in the upper 6 km depth (c). In all sections the earthquakes occurring +/- 1 km zone during the different periods are plotted (stars are M<4 earthquakes occurred during the 2018 eruptive period). Note the events clustering within the intruded dyke (sections b). Black circles indicate the intruding dyke and the PFF fault along which the M4.9 earthquake originated.
